# Supplementary material for: Pyruvate Dehydrogenase Kinase 1 inhibition mediated oxidative phosphorylation enhancement in cartilage promotes osteoarthritis progression
Source: BMC Musculoskelet Disord. 2023 Jul 20;24:597. doi: 10.1186/s12891-023-06585-6 (PMC10357736; doi:10.1186/s12891-023-06585-6)
Supplement: Supplementary file 1 — Additional file 1. [file 12891_2023_6585_MOESM1_ESM.zip › KEGG.Gsea.1653622432464/KEGG_PROTEASOME.html]

Details for gene set KEGG\_PROTEASOME[GSEA]

|  || Dataset | 55457\_collapsed\_to\_symbols.cls.cls #OA\_versus\_normal.cls.cls #OA\_versus\_normal\_repos |
| Phenotype | cls.cls#OA\_versus\_normal\_repos |
| Upregulated in class | OA |
| GeneSet | KEGG\_PROTEASOME |
| Enrichment Score (ES) | 0.40912735 |
| Normalized Enrichment Score (NES) | 1.5837702 |
| Nominal p-value | 0.021327015 |
| FDR q-value | 0.042223617 |
| FWER p-Value | 0.593 |
Table: GSEA Results Summary

  

Fig 1: Enrichment plot: KEGG\_PROTEASOME      
 Profile of the Running ES Score & Positions of GeneSet Members on the Rank Ordered List

  

| SYMBOL | TITLE | RANK IN GENE LIST | RANK METRIC SCORE | RUNNING ES | CORE ENRICHMENT || 1 | PSMB10 | proteasome 20S subunit beta 10 [Source:HGNC Symbol;Acc:HGNC:9538] | 39 | 1.011 | 0.0823 | Yes |
| 2 | PSMC2 | "proteasome 26S subunit, ATPase 2 [Source:HGNC Symbol;Acc:HGNC:9548]" | 559 | 0.638 | 0.0958 | Yes |
| 3 | PSMD4 | "proteasome 26S subunit ubiquitin receptor, non-ATPase 4 [Source:HGNC Symbol;Acc:HGNC:9561]" | 672 | 0.599 | 0.1377 | Yes |
| 4 | PSMB6 | proteasome 20S subunit beta 6 [Source:HGNC Symbol;Acc:HGNC:9543] | 1188 | 0.479 | 0.1380 | Yes |
| 5 | PSMB7 | proteasome 20S subunit beta 7 [Source:HGNC Symbol;Acc:HGNC:9544] | 1368 | 0.448 | 0.1619 | Yes |
| 6 | PSMD6 | "proteasome 26S subunit, non-ATPase 6 [Source:HGNC Symbol;Acc:HGNC:9564]" | 1370 | 0.448 | 0.1997 | Yes |
| 7 | PSMA2 | proteasome 20S subunit alpha 2 [Source:HGNC Symbol;Acc:HGNC:9531] | 1444 | 0.436 | 0.2308 | Yes |
| 8 | PSMB9 | proteasome 20S subunit beta 9 [Source:HGNC Symbol;Acc:HGNC:9546] | 1591 | 0.415 | 0.2545 | Yes |
| 9 | PSMB8 | proteasome 20S subunit beta 8 [Source:HGNC Symbol;Acc:HGNC:9545] | 1721 | 0.395 | 0.2778 | Yes |
| 10 | PSMB3 | proteasome 20S subunit beta 3 [Source:HGNC Symbol;Acc:HGNC:9540] | 1813 | 0.382 | 0.3029 | Yes |
| 11 | PSMC5 | "proteasome 26S subunit, ATPase 5 [Source:HGNC Symbol;Acc:HGNC:9552]" | 1987 | 0.362 | 0.3201 | Yes |
| 12 | PSMB2 | proteasome 20S subunit beta 2 [Source:HGNC Symbol;Acc:HGNC:9539] | 2015 | 0.360 | 0.3483 | Yes |
| 13 | SEM1 | SEM1 26S proteasome subunit [Source:HGNC Symbol;Acc:HGNC:10845] | 2029 | 0.358 | 0.3776 | Yes |
| 14 | PSMA5 | proteasome 20S subunit alpha 5 [Source:HGNC Symbol;Acc:HGNC:9534] | 2385 | 0.316 | 0.3766 | Yes |
| 15 | PSMA1 | proteasome 20S subunit alpha 1 [Source:HGNC Symbol;Acc:HGNC:9530] | 2822 | 0.267 | 0.3653 | Yes |
| 16 | PSMD14 | "proteasome 26S subunit, non-ATPase 14 [Source:HGNC Symbol;Acc:HGNC:16889]" | 3117 | 0.238 | 0.3625 | Yes |
| 17 | PSMF1 | proteasome inhibitor subunit 1 [Source:HGNC Symbol;Acc:HGNC:9571] | 3353 | 0.216 | 0.3625 | Yes |
| 18 | PSMA7 | proteasome 20S subunit alpha 7 [Source:HGNC Symbol;Acc:HGNC:9536] | 3366 | 0.215 | 0.3797 | Yes |
| 19 | PSME1 | proteasome activator subunit 1 [Source:HGNC Symbol;Acc:HGNC:9568] | 3388 | 0.213 | 0.3961 | Yes |
| 20 | PSMB1 | proteasome 20S subunit beta 1 [Source:HGNC Symbol;Acc:HGNC:9537] | 3531 | 0.199 | 0.4018 | Yes |
| 21 | PSMB4 | proteasome 20S subunit beta 4 [Source:HGNC Symbol;Acc:HGNC:9541] | 3860 | 0.169 | 0.3906 | Yes |
| 22 | PSMA3 | proteasome 20S subunit alpha 3 [Source:HGNC Symbol;Acc:HGNC:9532] | 3869 | 0.168 | 0.4041 | Yes |
| 23 | PSMC3 | "proteasome 26S subunit, ATPase 3 [Source:HGNC Symbol;Acc:HGNC:9549]" | 3977 | 0.158 | 0.4091 | Yes |
| 24 | PSMA4 | proteasome 20S subunit alpha 4 [Source:HGNC Symbol;Acc:HGNC:9533] | 4412 | 0.120 | 0.3855 | No |
| 25 | PSME2 | proteasome activator subunit 2 [Source:HGNC Symbol;Acc:HGNC:9569] | 4672 | 0.098 | 0.3737 | No |
| 26 | PSMD1 | "proteasome 26S subunit, non-ATPase 1 [Source:HGNC Symbol;Acc:HGNC:9554]" | 4751 | 0.091 | 0.3753 | No |
| 27 | PSMD2 | "proteasome 26S subunit ubiquitin receptor, non-ATPase 2 [Source:HGNC Symbol;Acc:HGNC:9559]" | 5083 | 0.066 | 0.3551 | No |
| 28 | PSMB5 | proteasome 20S subunit beta 5 [Source:HGNC Symbol;Acc:HGNC:9542] | 5586 | 0.025 | 0.3182 | No |
| 29 | PSMC6 | "proteasome 26S subunit, ATPase 6 [Source:HGNC Symbol;Acc:HGNC:9553]" | 5977 | -0.003 | 0.2881 | No |
| 30 | IFNG | interferon gamma [Source:HGNC Symbol;Acc:HGNC:5438] | 6410 | -0.036 | 0.2575 | No |
| 31 | PSMA6 | proteasome 20S subunit alpha 6 [Source:HGNC Symbol;Acc:HGNC:9535] | 6721 | -0.057 | 0.2382 | No |
| 32 | PSME3 | proteasome activator subunit 3 [Source:HGNC Symbol;Acc:HGNC:9570] | 6903 | -0.072 | 0.2302 | No |
| 33 | PSMD12 | "proteasome 26S subunit, non-ATPase 12 [Source:HGNC Symbol;Acc:HGNC:9557]" | 7845 | -0.144 | 0.1692 | No |
| 34 | PSMD7 | "proteasome 26S subunit, non-ATPase 7 [Source:HGNC Symbol;Acc:HGNC:9565]" | 7893 | -0.147 | 0.1780 | No |
| 35 | PSMD8 | "proteasome 26S subunit, non-ATPase 8 [Source:HGNC Symbol;Acc:HGNC:9566]" | 8004 | -0.155 | 0.1825 | No |
| 36 | PSMC1 | "proteasome 26S subunit, ATPase 1 [Source:HGNC Symbol;Acc:HGNC:9547]" | 8204 | -0.172 | 0.1815 | No |
| 37 | POMP | proteasome maturation protein [Source:HGNC Symbol;Acc:HGNC:20330] | 8244 | -0.174 | 0.1932 | No |
| 38 | PSMC4 | "proteasome 26S subunit, ATPase 4 [Source:HGNC Symbol;Acc:HGNC:9551]" | 8886 | -0.224 | 0.1622 | No |
| 39 | PSMD11 | "proteasome 26S subunit, non-ATPase 11 [Source:HGNC Symbol;Acc:HGNC:9556]" | 9749 | -0.296 | 0.1202 | No |
| 40 | PSME4 | proteasome activator subunit 4 [Source:HGNC Symbol;Acc:HGNC:20635] | 10694 | -0.392 | 0.0798 | No |
| 41 | PSMD13 | "proteasome 26S subunit, non-ATPase 13 [Source:HGNC Symbol;Acc:HGNC:9558]" | 10797 | -0.405 | 0.1061 | No |
| 42 | PSMD3 | "proteasome 26S subunit, non-ATPase 3 [Source:HGNC Symbol;Acc:HGNC:9560]" | 12307 | -0.679 | 0.0461 | No |
Table: GSEA details [plain text format]

  

Fig 2: KEGG\_PROTEASOME      
 Blue-Pink O' Gram in the Space of the Analyzed GeneSet

  

Fig 3: KEGG\_PROTEASOME: Random ES distribution      
 Gene set null distribution of ES for **KEGG\_PROTEASOME**
